# Outcome measures in older persons with acquired joint contractures: a systematic review and content analysis using the ICF (International Classification of Functioning, Disability and Health) as a reference

**DOI:** 10.1186/s12877-016-0213-6

**Published:** 2016-02-09

**Authors:** Gabriele Bartoszek, Uli Fischer, Martin Müller, Ralf Strobl, Eva Grill, Stephan Nadolny, Gabriele Meyer

**Affiliations:** Faculty of Health, School of Nursing Science, Witten/Herdecke University, Witten, Germany; Institute for Health and Nursing Science, Martin Luther University, Halle-Wittenberg, Germany; Institute for Medical Information Processing, Biometrics and Epidemiology, Ludwig-Maximilians-Universität München, Munich, Germany; German Center for Vertigo and Balance Disorders, Ludwig-Maximilians-Universität München, Munich, Germany

**Keywords:** Joint contracture, Aged, Outcome, Assessment, Geriatric rehabilitation, Nursing homes

## Abstract

**Background:**

Joint contractures are a common health problem in older persons with significant impact on activities of daily living. We aimed to retrieve outcome measures applied in studies on older persons with joint contractures and to identify and categorise the concepts contained in these outcome measures using the ICF (International Classification of Functioning, Disability and Health) as a reference.

**Methods:**

Electronic searches of Medline, EMBASE, CINAHL, Pedro and the Cochrane Library were conducted (1/2002-8/2012). We included studies in the geriatric rehabilitation and nursing home settings with participants aged ≥ 65 years and with acquired joint contractures. Two independent reviewers extracted the outcome measures and transferred them to concepts using predefined conceptual frameworks. Concepts were subsequently linked to the ICF categories.

**Results:**

From the 1057 abstracts retrieved, 60 studies met the inclusion criteria. We identified 52 single outcome measures and 24 standardised assessment instruments. A total of 1353 concepts were revealed from the outcome measures; 96.2 % could be linked to 50 ICF categories in the 2nd level; 3.8 % were not categorised. Fourteen of the 50 categories (28 %) belonged to the component Body Functions, 4 (8 %) to the component Body Structures, 26 (52 %) to the component Activities and Participation, and 6 (12 %) to the component Environmental Factors.

**Conclusions:**

The ICF is a valuable reference for identifying and quantifying the concepts of outcome measures on joint contractures in older people. The revealed ICF categories remain to be validated in populations with joint contractures in terms of clinical relevance and personal impact.

**Electronic supplementary material:**

The online version of this article (doi:10.1186/s12877-016-0213-6) contains supplementary material, which is available to authorized users.

## Background

Joint contractures are characterised by a lack of full range of motion (ROM) of a joint and go along with deformity, disuse and pain. Joint contractures in upper limbs may result in inability to dress or eat independently, while contractures in lower limbs may cause instability, inability to walk and higher risk of bed confinement [1–3]. Joint contractures are recognised in the geriatric community as a disabling complication by frail older persons, particularly in residents of nursing homes [3–5]. International studies indicate a prevalence of joint contractures in older persons ranging between 20 % and 80 % [6–8]. This wide variation is due to heterogeneous definitions of joint contracture, different diagnostic criteria and data collection methods, different settings, sample sizes and participants’ characteristics [1, 9].

In clinical settings, joint contractures are usually assessed by measuring the range of motion [10]. A variety of other functional measures is currently used for the assessment and evaluation of geriatric patients [10]. However, the impact of contractures on functioning, quality of life, and the ability to participate in everyday life seem to be assessed less often.

The International Classification of Functioning, Disability, and Health (ICF) [11] provides a useful framework for health outcome measurement in older persons [12]. The ICF can be understood as the operationalization of health and represents the outcome of the interaction between a person’s health condition and his/her contextual factors [13]. The ICF is divided into two parts, with two components each. Part 1 covers Functioning and Disability and includes the components Body Functions (b) and Body Structures (s) as well as Activities and Participation (d). Part 2 covers Contextual Factors and contains the components Environmental Factors (e) and Personal Factors (pf) [11]. The review presented herein is a part of a broader project [14] aimed at deriving a standard set according to the methods recommended by the WHO for ICF Core Set development [13, 15].

The aims of our review were 1) to retrieve outcome measures applied in studies focusing on older persons with acquired joint contractures and 2) to identify and categorise the concepts contained in these outcome measures using the ICF as a reference.

## Methods

### Literature search and study selection

A systematic literature search was conducted in the following databases: Medline via PubMed, EMBASE, CINAHL, Pedro and the Cochrane Library. We included studies that had been carried out in geriatric rehabilitation hospitals or nursing homes. Participants had to be 65 years or older and to have acquired joint contractures. Studies dealing with congenital or genetic joint contractures were excluded. Three groups of search terms were combined (text words and MeSH terms, if available): 1) contracture, joint contracture; 2) elderly, old people, age, geriatric; and 3) geriatric care and nursing home. The search was limited to papers in German and English published between January 2002 and August 2012. The time limitation was applied since we aimed to identify outcome measures that are used in contemporary research [13]. The search strategy for PubMed is displayed in Table 1.

**Table 1:** Complete search strategy – PubMed

(((“Contracture” [Mesh] OR “joint contracture* ”) AND (elderly OR geriatric OR aged OR “older person*” OR “old people”) NOT dupuytren) NOT (Meta-Analysis[ptyp] OR Review [ptyp] OR Case Reports [ptyp]))) Filters: published in the last 10 years; English; German.	

Since we wanted to draw a comprehensive picture of the content of outcome measures used in studies focusing on joint contracture outcomes in older persons, we decided to include randomised controlled trials, as well as controlled clinical trials, cohort studies, cross-sectional studies and case–control studies.

The titles and abstracts of citations retrieved were screened and eligible full text articles were assessed by two independent reviewers. Results were compared and disagreement solved by discussion. A third reviewer was consulted when required.

### Data extraction and ICF linking procedure

In a first step, the two reviewers extracted the outcome measures applied in the studies and the descriptive study characteristics, using a standardised electronic form. We included both standardised assessment instruments, like the Knee Society Score [16], and single outcome measures, such as joint range of motion measurement and specific clinical tests such as x-ray. Assessment instruments were data extracted on the item level [13]. If the assessment was just mentioned but not described in detail in the retrieved study, we obtained it by reference checking, searching in books on clinical measures, and through internet search [17].

In a second step, the concepts that are contained in the assessment instrument items and single outcome measures were extracted [13]. For example, the item “heavy household duties” of the Western Ontario McMaster University Osteoarthritis Index [18] was conceptualized as “housework”.

In a third step, the concepts of the outcome measures were linked to the ICF categories using established linking rules by trained researchers [13, 19]. For example, the concept “housework” corresponds with the ICF category “Doing housework” (d640)”. Personal Factors are not covered by the ICF and could therefore not be linked, e.g. concepts on patient satisfaction or coping [13].

If a concept was judged as too general to allow a decision on the linking to a specific ICF chapter, domain or category, the concept was considered as “not defined” [19], e.g. “any activity”. At every step, the two independent reviewers (authors GB, SN) compared their results. Initial disagreement was solved by consensus. If there was no consensus, a third researcher (UF) was consulted. For quality assurance purposes, the reviewers attended a two-day training course provided by a senior ICF expert (MM) in preparation for the linking procedure. The senior expert supervised the entire process.

### Data analysis

Absolute and relative frequencies of the standardised outcome assessment instruments and single outcomes were calculated. Only assessment instruments and single outcome measures used in at least two different studies are reported in this manuscript.

Relative frequencies of ICF categories and 95 % confidence intervals were calculated. An ICF category that emerged more than once in a publication was counted only one time [13].

Only ICF categories referring to concepts measured in more than 5 % of the studies are reported in this manuscript. The structure of the ICF is displayed in the Additional file 1: The four major components (Body Functions, Body Structures, Activity and Participation, Environmental Factors) each have a number of sub-classifications, called Chapters (first level), which again are sub-classified in Categories (second level). Each second-level category has sub-categories (third level), which in turn have sub-categories (fourth level). The example at the bottom of the chart shows the levels, into which Chapter b2 (Body Functions) is divided.

ICF categories are here presented at the 2nd level. If a concept had been linked to a 3rd or 4th level ICF category, i.e. a level with more detail, the corresponding 2nd level category is reported. Due to the hierarchical structure of the ICF and its codes, a category of a higher level of detail can be transferred to the category with a lower level of detail by deleting the appropriate number of digits of the ICF code (e.g. the 3^rd^ level category “Manipulating” (d4402) can be transferred to the 2^nd^ level category “Fine hand use” (d440)).

## Results

**Fig. 1:**
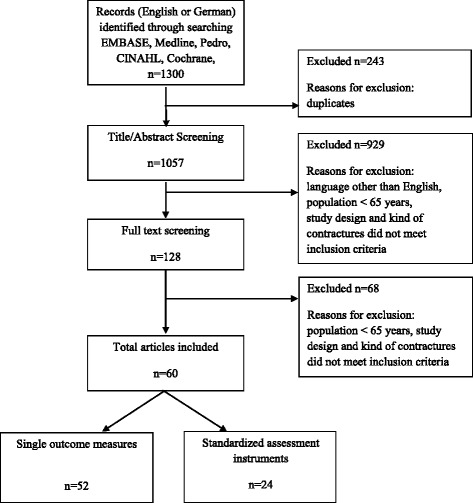
Flow chart showing the search process and the inclusion of studies in the review

Initially, a total of 1057 publications were identified; *n* = 60 met the inclusion criteria and were included in the review. Figure 1 displays the flow of the literature search.

The majority of included publications (*n* = 52, 87 %) were authored by medical scientists and physiotherapists, *n* = 6 (10 %) focussed on the acute and post-acute setting. Study participants suffered predominately from musculoskeletal (*n* = 51, 85 %) or neurological diseases (*n* = 7, 12 %). A total of 55 studies dealt with an intervention, either invasive (surgery or injections: *n* = 32), non-invasive (*n* = 20, e.g. splint, exercise programmes) or both invasive and non-invasive interventions (*n* = 3, e.g. injections as preparation for a stretching programme). The remaining five studies featured diagnostic procedures. Two studies [5, 6] addressed the nursing home setting and either estimated the prevalence of major joint contractures by a proxy assessment for persons with cognitive impairment [5] or measured the effect of a restorative care approach on the prevention of joint contractures [6].

The included studies covered a range of study designs, i.e. randomised controlled trials (*n* = 12), controlled clinical trials (*n* = 2), cross-sectional studies (*n* = 29), and cohort studies (*n* = 17).

In total, we identified 24 standardised assessment instruments and 52 single outcome measures. Table 2 displays the standardised outcome assessment instruments and Table 3 the single outcome measures that were reported in at least two different studies (*n* = 12 and *n* = 19, respectively). Throughout the 60 studies the most often used standardised assessment instruments were the Knee Society Score (KSS, *n* = 21) [16], followed by the Hospital for Special Surgery Score (HSS, *n* = 8) [20], the 3D Gait analysis (*n* = 5) [21], the Western Ontario McMaster University Osteoarthritis Index Scales (WOMAC, *n* = 4) [18], and the Motor Assessment Scale (MAS, *n* = 4) [22]. All other standardised assessment instruments identified were applied in 5 % or less of the included studies. The five most often reported single outcome measures throughout the 60 studies were range of motion of the knee (*n* = 34), x-ray examination of the knee (*n* = 19), and pain score for the knee (*n* = 10), followed by pain score for the shoulder (*n* = 5) and range of motion of the shoulder (*n* = 5).

**Table 2:** Standardized outcome assessment instruments used in the 60 studies included

Outcome assessment instrument^a^	No. (%)
Knee Society Score [19]	21 (35)
Hospital for Special Surgery Score [20]	8 (13)
3D Gait analysis [21]	5 (8)
Western Ontario McMaster University Osteoarthritis Index Scales [18]	4 (7)
Motor Assessment Scale [22]	4 (7)
Barthel Index [28]	3 (5)
The Action Research Arm Test [29]	2 (3)
Tardieu Scale [30]	2 (3)
Short Form Health Survey, SF-12 [31]	2 (3)
Modified Ashworth Scale [32]	2 (3)
Mayo Elbow Performance Index [33]	2 (3)
Disabilities of the Arm, Shoulder and Hand [34]	2 (3)

^a^Only instruments that were used in at least two different studies are displayed

Values are absolute numbers (percentages)

**Table 3:** Single outcomes used in the 60 studies included

Measurement^a^	No. (%)
Range of motion (knee)	34 (57)
X-ray (knee)	19 (32)
Pain score (knee)	10 (17)
Subjects were asked to first stand and then walk along a 10 m walkway	5 (8)
Range of motion (shoulder)	5 (8)
Pain score (shoulder)	5 (8)
Stability of joint function (stabilometry)	4 (7)
Pain score (upper limb)	4 (7)
Hand grip strength	3 (5)
Range of motion (hip)	3 (5)
Strength of the knee extensors	3 (5)
Chair rise test	2 (3)
Muscle power (shoulder)	2 (3)
Range of motion (ankle)	2 (3)
Range of motion (finger/wrist)	2 (3)
Range of motion (lower limb, matching task)	2 (3)
X-ray (elbow)	2 (3)
X-ray (hip)	2 (3)
X-ray (shoulder)	2 (3)

^a^Only single outcomes that were used in at least two different studies are displayed

Values are absolute numbers (percentages)

A total of 1353 concepts were revealed from the outcome measures. We were able to link 96.2 % of these concepts to ICF categories; 2.5 % (*n* = 34 concepts) were considered as “not defined” and 1.3 % (*n* = 18) as Personal Factors. The concepts were linked to 155 ICF categories. Five ICF categories (3.2 %) were linked to the 1st level of the ICF, *n* = 52 (33.5 %) to 2nd level ICF categories, *n* = 88 (56.8 %) to 3rd level ICF categories and *n* = 10 (6.5 %) to 4th level ICF categories. The Tables 4, 5, 6 and 7 display the 2nd level ICF categories (*n* = 50) derived from the concepts of the standardised outcome assessment instruments and single outcomes. There were five ICF categories which were represented most frequently (>50 % of the studies) and 21 ICF categories frequently (>10 % of the studies). Two of the five ICF categories are part of the component Body Functions (Table 4): “Mobility of joint functions” (b710) (represented in 98 % of included studies) and “Sensation of pain” (b280) (70 %). The other three ICF categories most frequently represented were “Structure of lower extremity” (s750) (72 %) belonging to the component Body Structures (Table 5), “Walking” (d450) (65 %) and “Moving around” (d455) (53 %) from the component Activities and Participation (Table 6). Six Environmental Factors were categorised (Table 7), two of them – “Products and technology for personal indoor and outdoor mobility and transportation” (e120) (45 %) and “Design, construction and building products and technology of buildings for private use” (e155) (37 %) – were frequently represented ICF categories.

**Table 4:** Relative frequency of 2nd level ICF categories. Component body functions (b)

ICF code	ICF category	% (95 % CI)
	ICF chapter mental function	
b134	Sleep functions	8 (2.8 to 18.4)
b152	Emotional functions	8 (2.8 to 18.4)
b235	Vestibular functions	5 (1 to 13.9)
	ICF Chapter Sensory Function and Pain	
b280	Sensation of pain	70 (56.8 to 81.2)
	ICF Chapter Function of Digestive, Metabolic and Endocrine Systems	
b525	Defecation functions	7 (1.8 to 16.2)
	ICF Chapter Genitourinary and Reproductive Function	
b620	Urination functions	7 (1.8 to 16.2)
	ICF Chapter Neuromusculoskeletal and Movement-related Function	
b710	Mobility of joint functions	98 (91.1 to 100)
b715	Stability of joint functions	47 (33.7 to 60)
b720	Mobility of bone functions	8 (2.8 to 18.4)
b730	Muscle power functions	33 (21.7 to 46.7)
b735	Muscle tone functions	12 (4.8 to 22.6)
b755	Involuntary movement reaction functions	5 (1 to 13.9)
b770	Gait pattern functions	13 (5.9 to 24.6)
b780	Sensations related to muscles and movement functions	7 (1.8 to 16.2)

Values are percentages (95 % CI); the denominator is the number of studies included (*n* = 60). ICF categories referring to concepts measured in more than 5 % of the studies are reported

**Table 5:** Relative frequency of 2nd level ICF categories. Component body structures (s)

ICF code	ICF category	% (95 % CI)
	ICF chapter structure related to movement	
s720	Structure of shoulder region	13 (5.9 to 24.6)
s730	Structure of upper extremity	22 (12.1 to 34.2)
s740	Structure of pelvic region	8 (2.8 to 18.4)
s750	Structure of lower extremity	72 (58.6 to 82.5)

Values are percentages (95 % CI); the denominator is the number of studies included (*n* = 60). ICF categories referring to concepts measured in more than 5 % of the studies are reported

**Table 6:** Relative frequency of 2nd level ICF categories. Component activities and participation (d)

ICF code	ICF category	% (95 % CI)
	ICF chapter general tasks and demands	
d230	Carrying out daily routine	7 (1.8 to 16.2)
	ICF Chapter Mobility	
d410	Changing basic body position	30 (18.8 to 43.2)
d415	Maintaining a body position	17 (8.3 to 28.5)
d420	Transferring oneself	22 (12.1 to 34.2)
d430	Lifting and carrying objects	8 (2.8 to 18.4)
d440	Fine hand use	13 (5.9 to 24.6)
d445	Hand and arm use	18 (9.5 to 30.4)
d450	Walking	65 (51.6 to 76.9)
d455	Moving around	53 (40 to 66.3)
d465	Moving around using equipment	8 (2.8 to 18.4)
d470	Using transportation	13 (5.9 to 24.6)
d475	Driving	8 (2.8 to 18.4)
	ICF Chapter Self-care	
d510	Washing oneself	20 (10.8 to 32.3)
d520	Caring for body parts	10 (3.8 to 20.5)
d530	Toileting	15 (7.1 to 26.6)
d540	Dressing	20 (10.8 to 32.3)
d550	Eating	12 (4.8 to 22.6)
d560	Drinking	5 (1 to 13.9)
d570	Looking after one’s health	7 (1.8 to 16.2)
	ICF Chapter Domestic Life	
d620	Acquisition of goods and services	8 (2.8 to 18.4)
d640	Doing housework	13 (5.9 to 24.6)
d650	Caring for household objects	7 (1.8 to 16.2)
	ICF Chapter Interpersonal Interactions and Relationships	
d770	Intimate relationships	7 (1.8 to 16.2)
	ICF Chapter Major Life Areas	
d850	Remunerative employment	7 (1.8 to 16.2)
	ICF Chapter Community, Social and Civic life	
d920	Recreation and leisure	12 (4.8 to 22.6)
d930	Religion and spirituality	7 (1.8 to 16.2)

Values are percentages (95 % CI); the denominator is the number of studies included (*n* = 60). ICF categories referring to concepts measured in more than 5 % of the studies are reported

**Table 7:** Relative frequency of 2nd level ICF categories. Environment (e)

ICF code	ICF category	% (95 % CI)
	ICF Chapter Support and Relationships	
e310	Immediate family	7 (1.8 to 16.2)
e315	Extended family	7 (1.8 to 16.2)
e320	Friends	7 (1.8 to 16.2)
e399	Support and relationships, unspecified	20 (10.8 to 32.3)
	ICF Chapter Products and Technology	
e120	Products and technology for personal indoor and outdoor mobility and transportation	45 (32.1 to 58.4)
e155	Design, construction and building products and technology of buildings for private use	37 (24.6 to 50.1)

Values are percentages (95 % CI); the denominator is the number of studies included (*n* = 60). ICF categories referring to concepts measured in more than 5 % of the studies are reported

## Discussion

This systematic review provides a detailed analysis of the content of outcome measures used in research dealing with joint contractures in older persons. We analysed 60 publications reporting on 52 single outcome measures and 24 standard assessment instruments revealing 1353 concepts. These concepts were linked to 50 2nd level ICF categories.

The most often linked categories emerged from the three assessment instruments KSS, HSS and WOMAC. These are used predominately in surgical and orthopedic evaluation [23, 24], but they address limitations in activities of daily living insufficiently and do not even address social participation.

Even though a relevant number of ICF categories (*n* = 26) belong to the component Activities and Participation, the chapter “Mobility” (*n* = 12) and “Self-care” (*n* = 7) are dominant and other limitations experienced by persons affected by joint contractures are not addressed [25], e.g. “Remunerative employment ”, “Economic self-sufficiency” or “Informal social relationships”.

Three out of five most often linked ICF categories (Body Function: “Sensation of pain”; Activities and Participation: “Walking” and “Moving around”) have earlier been shown as highly predictive for the development of a joint contracture [1–3, 5–8].

Compared to the ICF components Body Structures and Activities Participation, a relatively low number of linked categories (*n* = 6) belonged to the component Environmental Factors. Three of these six categories of Environmental Factors were found frequently in our review (in 20 %, 37 %, and 45 % of the 60 studies analysed), indicating that at least some contextual factors are considered relevant for functioning of persons with joint contractures. Since our recent qualitative interviews draw the attention to the major role of mobility for daily life of older persons with joint contractures [25], modelling of future joint contracture outcomes should take environmental factors into account. The importance of facilitators of walking and moving, such as walking aids and creation of barrier-free buildings has been pointed out in former research dealing with joint contractures [25–27].

Our study has potential limitations. Linking concepts of outcome measures to ICF categories is not simple and straightforward. Recent linking exercises, however, have demonstrated that it is possible to examine and compare the content of measures based on the ICF framework [13].

We did not review the psychometric properties of the outcome measures identified. However, this systematic review was solely dedicated to the description of outcome measures used in recent research as the first step in the generation of an ICF standard set on joint contractures. It was not our intention to critically appraise existing assessment instruments and single outcome measures in order to decide which outcome measure should be used.

## Conclusion

The revealed ICF categories remain to be validated in terms of clinical relevance and personal impact in populations affected by joint contractures. Our consecutive steps towards ICF standard set development will be reported elsewhere.

## Additional file

Additional file 1:
**Structure of the International Classification of Functioning, Disability and Health (ICF)**. (DOC 67 kb)
