# Supplementary figures and images for: Outcome measures in older persons with acquired joint contractures: a systematic review and content analysis using the ICF (International Classification of Functioning, Disability and Health) as a reference
Source: BMC Geriatr. 2016 Feb 9;16:40. doi: 10.1186/s12877-016-0213-6 (PMC4748463; doi:10.1186/s12877-016-0213-6)

**Additional File**


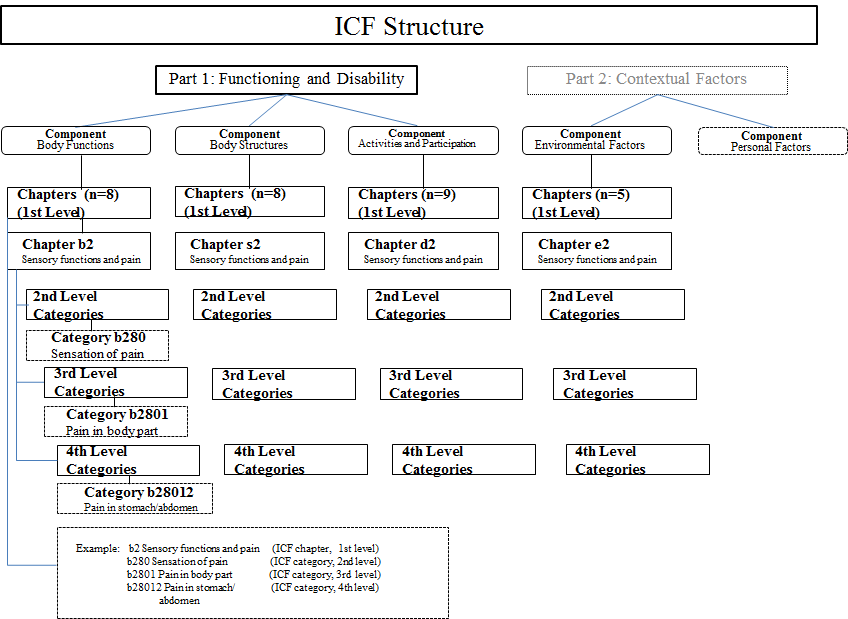

Supplement: Additional file 1: — Structure of the International Classification of Functioning, Disability and Health (ICF). (DOC 67 kb) [file 12877_2016_213_MOESM1_ESM.doc]
